# A Quantitative Study of Network Robustness in Resting-State fMRI in Young and Elder Adults

**DOI:** 10.3389/fnagi.2015.00256

**Published:** 2016-02-03

**Authors:** Jaime Gomez-Ramirez, Yujie Li, Qiong Wu, Jinglong Wu

**Affiliations:** ^1^Department of Neuroscience and Mental Health, The Hospital for Sick Children, University of Toronto, Toronto, ON, Canada; ^2^Key Laboratory of Adolescent Cyberpsychology and Behavior (CCNU), Ministry of Education, Wuhan, China; ^3^School of Psychology, Central China Normal University, Wuhan, China; ^4^Biomedical Engineering Laboratory, Okayama University, Okayama, Japan; ^5^Intelligent Robotics Institute, School of Mechatronics Engineering, Beijing Institute of Technology, Beijing, China

**Keywords:** normal aging, resting-state fMRI, network robustness, network efficiency, network degeneration hypothesis

## Abstract

Brain connectivity analysis has shown great promise in understanding how aging affects functional connectivity; however, an explanatory framework to study healthy aging in terms of network efficiency is still missing. Here, we study network robustness, i.e., resilience to perturbations, in resting-state functional connectivity networks (rs-fMRI) in young and elder subjects. We apply analytic measures of network communication efficiency in the human brain to investigate the compensatory mechanisms elicited in aging. Specifically, we quantify the effect of “lesioning” (node canceling) of either single regions of interest (ROI) or whole networks on global connectivity metrics (i.e., efficiency). We find that young individuals are more resilient than old ones to random “lesioning” of brain areas; global network efficiency is over 3 times lower in older subjects relative to younger subjects. On the other hand, the “lesioning” of central and limbic structures in young subjects yield a larger efficiency loss than in older individuals. Overall, our study shows a more idiosyncratic response to specific brain network “lesioning” in elder compared to young subjects, and that young adults are more resilient to random deletion of single nodes compared to old adults.

## 1 Introduction

The concept of brain reserve has its origins in the experimental observation of the mismatch between disease-related changes in the brain and the clinical manifestation of those changes. For example, postmortem analysis of people with Alzheimer’s disease showed a non-negligible number of individuals with fewer clinical symptoms than the pathological features suggested (Katzman et al., 1988). These individuals have heavier brains containing more neurons or a greater “reserve” that could help fight cognitive decline associated with brain damage, e.g., neuronal loss (Crystal et al., 1988; Guo et al., 2013). According to the brain reserve hypothesis, clinical expression of pathologies and aging effects can be effectively attenuated or delayed in time in those individuals with more resilient or larger reserve brains (Mortimer, 1997). Cognitive reserve refers to the ability to alter brain reserve through several means, including diet, education, and lifestyle (Whalley et al., 2004; Stern, 2012; Mora, 2013; Bozzali et al., 2015; Freret et al., 2015). The concept of reserve or resilience can help to explain the neuroprotective effects triggered by brain changes and is supported by structural (Solé-Padullés et al., 2009; Bartrés-Faz and Arenaza-Urquijo, 2011) and functional imaging studies (Stern et al., 2005; Jellinger and Attems, 2013).

A theory of the mechanisms underlying the neuroprotective effect of brain resilience in the face of brain changes, e.g., aging, is still missing. Here, we try to understand quantitatively the relationship between resilience and successful aging. Aging is a complex physiological process with multiple temporal and spatial scales, and it is unrealistic to expect any all encompassing predictive model of aging. Nevertheless, a common finding is that the older subjects present reduced functional connectivity compared to young adults (Andrews-Hanna et al., 2007; Sambataro et al., 2010; St Jacques et al., 2010; Campbell et al., 2013). We will try to replicate this finding and go beyond, identifying the brain networks that when disconnected from the rest, result in dramatic/mild efficiency loss in transmitting information.

We explore the hypothesis that normal aging is associated with changes in network efficiency. Transport network efficiency measures have been used to study the relationship between structural and resting-state functional connectivity (Goni et al., 2014). The effects of lesioning in white matter connections can be studied via the simulation of the removal of individual connections from the connectome. Irimia and Van Horn (2014)’s report using this technique has been able to delineate “a core scaffold” or white matter network connections that when disrupted trigger dramatic changes in the overall organization of the human connectome. However, a systematic study of the effects of simulated lesioning in rs-fMRI is still missing. In this paper, we try to fill this gap, providing efficiency and robustness measures to quantify the impact of simulated lesioning. We investigate the hemispheric asymmetry reduction hypothesis (Cabeza et al., 2002), studying the effects of lesioning hemispheres separately in older compared to young adults.

## 2 Materials and Methods

### 2.1 Data Acquisition

Forty-two healthy volunteers separated in two groups, twenty-three healthy young volunteers (ages 21–32; mean 22.7; SD 2.48; male/female 23/0) and 19 healthy older volunteers (ages 60–78; mean 66.5; SD 4.93; male/female 16/3; MMSE score 29.5 ± 0.1) took part in the fMRI experiment. All subjects had normal or corrected-to-normal vision, and all the participants in both age groups have not been diagnosed with mild cognitive impairment or psychiatric or neurological disorders. The study was approved by the ethics committee of Okayama University, and written informed consent was obtained before the study. All subjects were imaged using a 1.5-T Philips scanner vision whole-body MRI system (Okayama University Hospital, Okayama, Japan), which was equipped with a head coil. Functional MR images were acquired during rest when subjects were instructed to keep their eyes closed and not to think of anything in particular. The imaging area consisted of 32 functional gradient-echo planar imaging (EPI) axial slices (voxel size = 3 mm × 3 mm × 4 mm, TR = 3000 ms, TE = 50 ms, FA = 90°, acquisition matrix = 80 × 79, FOV = 240 mm × 240 mm, slice thickness = 4 mm, gap = 0.5 mm) that were used to obtain T2*-weighted fMRI images in the axial plane. We obtained 176 functional volumes and excluded the first 4 scans from analysis. After the EPI scan, a T1-weighted 3D magnetization-prepared rapid acquisition gradient echo (MP-RAGE) sequence was acquired (TR = 9.4 ms, TE = 4.6 ms, FA = 10°, acquisition matrix = 240 × 240, voxel size = 1 mm × 1 mm × 1 mm, 200 contiguous axial slices).

### 2.2 Data Preprocessing

Data were preprocessed using Statistical Parametric Mapping software SPM8^1^ and REST v1.7.^2^ To correct for differences in slice acquisition time, all images were synchronized to the middle slice. Subsequently, images were spatially realigned to the first volume due to head motion. None of the subjects in both conditions had head movements exceeding 2.5 mm on any axis or rotations >2.5°. After the correction, the imaging data were normalized to the Montreal Neurological Institute (MNI) EPI template supplied with SPM8 (resampled to 2 mm × 2 mm × 2 mm voxels).^3^ In order to avoid introducing artificially local spatial correlation, the normalized images were not smoothed. Finally, the resulting data were temporally band-pass filtered (0.01–0.08 Hz) to reduce the effects of low-frequency drifts and high-frequency physiological noises (Jiao et al., 2011).

### 2.3 Anatomical Parcellation

Before whole-brain parcellation, several sources of spurious variance including the estimated head motion parameters, the global brain signal, and the average time series in the cerebrospinal fluid and white matter regions were removed from the data through linear regression. It ought to be noted that Murphy et al. (2009) have pointed out that global signal removal may artificially introduce anticorrelated networks. The effect of the removal of the global signal on resting-state correlation maps have been examined by Fox et al. (2009), reaching to the conclusion that several characteristics of anticorrelated networks are not attributable to global signal removal and therefore suggesting a biological basis for those anticorrelations.

The fMRI data were parcellated into 90 regions using the automated anatomical labeling template (AAL) (Tzourio-Mazoyer et al., 2002). For each subject, the mean time series of each region was obtained by simply averaging the time series of all voxels within that region.

### 2.4 Brain Network Construction

**Figure 1:**
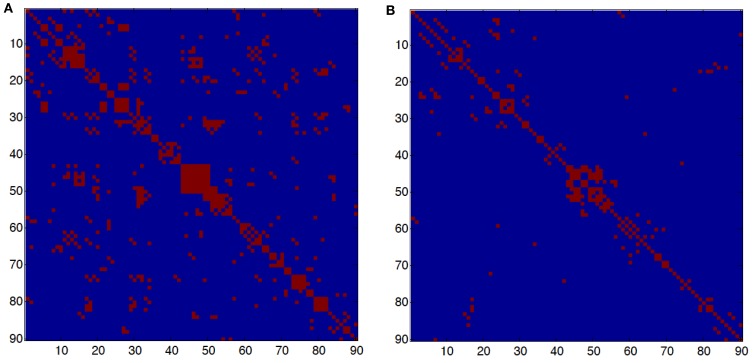
**(A)** Adjacency matrix in young subjects. **(B)** Adjacency matrix in old subjects. The red dots represent connections between two nodes or brain regions. An element *i*, *j* of the adjacency matrix is *M*(*i, j*) = 1 if there is a significant correlation between brain regions *i* and *j* and *M*(*i, j*) = 0, otherwise. The number of edges in the young group is 718 and in the old group is 308; the average degree connectivity is 8.97 and 4.42, respectively.

To measure the functional connectivity among regions, we calculated the Pearson correlation coefficients between any possible pair of regional time series and then obtained a temporal correlation matrix (90 × 90) for each subject. We applied Fisher’s r-to-z transformation to improve the normality of the correlation matrix. Then, two-tailed one-sample t-tests were performed for all the possible $4005=\frac{90\times 89}{2}$ pairwise correlations across subjects to examine whether each interregional correlation significantly differed from zero. A Bonferroni–Holm correction for multiple comparisons was further used to threshold the correlation matrix into the adjacency matrix M as shown in Figure 1. Finally, an undirected binary graph was acquired in which nodes represent brain regions and edges represent links between regions.

### 2.5 Information Efficiency

A quantitative understanding of network robustness, that is, functional network invariance under perturbation, can shed light on the properties that mediate in developmental, aging, and pathological processes in the human brain. In essence, robustness measures the capacity of the network to perform the same function before and after a perturbation. Perturbations are events, internal or external, that elicit a change in the network configuration. Possible perturbations are the obliteration of one or more nodes and changes in the connectivity between nodes.

The efficiency of a network is a network centrality measure that quantifies the network’s reliability in transmitting information. Latora and Marchiori (2001) proposed a measure of network efficiency, which is defined as the efficiency in transmitting information between any two nodes (*i*, *j*) in a graph G as the inverse of the shortest path that connects them.

(1) $$\varepsilon_{\mathrm{ij}}=\frac{1}{d_{\mathrm{ij}}}$$

where *d_ij_* is the shortest path length or the geodesic distance between nodes *i* and *j*. Note that when there is no path that connects the nodes i and j, *d_ij_* = ∞, and the efficiency in the communication of the two nodes is zero, *ε_ij_* = 0.

The efficiency of the network G, Σ(*G*), is then calculated as the average of the efficiency between any two nodes *ε_ij_*

(2) Σ(G)=∑i≠j εijN(N−1)=1N(N−1)1∑i≠j dij

where *N* is the number of nodes.

We can calculate the *information centrality C* of any node i in a network G as the variation in the network efficiency caused by the removal of the edges incident in i. Thus, the information centrality of a node i, *C_i_*, is the difference between the efficiency of the original network G with N nodes and E edges, *G*(*N,E*), and the efficiency of the resulting graph *G*(*N* − *i*, *E* − *k_i_*) with *N* − *i* nodes and *E* − *k_i_* edges, where *k_i_* denotes the set of edges incident to node i. The centrality of a node is a normalized measure of the loss in network efficiency, caused by the isolation of a node in G. Thus, the centrality of node i or the *efficiency loss* after the disconnection of node i is

(3) $$C_{i}=\frac{\Sigma \left(G\left(N,E\right)\right)-\Sigma \left(G\left(N-i,E-k_{i}\right)\right)}{\Sigma \left(G\left(N,E\right)\right)}$$

From equation (3), a network *G* is considered to be robust to a perturbation if the network efficiency, Σ(*G*), stays close to the original value after a perturbation. Ideally, Σ(*G*(*N*, *E*)) = Σ(*G*(*N* − *i*, *E* − *k_i_*)) with efficiency loss or centrality of node i equals to 0.

By the same token, the information centrality of a set of nodes *S* or the efficiency loss upon the removal of *S* can be calculated as the normalized measure of the loss in network efficiency caused by the isolation of a set of nodes S in G.

(4) $$C_{S}=\frac{\Sigma \left(G\left(N,E\right)\right)-\Sigma \left(G\left(N-S,E-k_{S}\right)\right)}{\Sigma \left(G\left(N,E\right)\right)}$$

## 3 Results

The global network efficiency for unperturbed networks as defined in equation (2) is 0.3678 for young subjects and 0.1144 for elder subjects. Thus, young subjects’ connectivity network is slightly more than three times more efficient in terms of the shortest path distance between any two nodes as calculated in equation (2).

In order to obtain the efficiency measures described in equations (3) and (4), we perturb the resting-state network in the following three ways: first, using random single-node deletion (Section 3.1); second, targeting specific networks of interest (Section 3.2); and third, the efficiency loss after lesioning edges (Section 3.3).

### 3.1 Efficiency after Single-Node Lesioning

Here, we build a population of networks created by the systematic lesioning of single nodes. The population of perturbations *P* that result from the systematic deletion of all nodes in all possible combinations has as many networks as

$$\left|P\right|=\sum_{i=1}^{N}C\left(N,i\right)=\frac{N!}{\left(i!\right)\left(N-i\right)!}$$

For example, the population of networks that result from the deletion of one single node has 90 networks

$$\sum_{i=1}^{1}C\left(90,i\right)=\frac{90!}{\left(1!\right)\left(90-1\right)!}=90$$

Similarly, the number of perturbed networks obtained by deleting two nodes in all possible ways contains 4005 networks

$$\sum_{i=1}^{2}C\left(90,i\right)=\frac{90!}{\left(2!\right)\left(90-2\right)!}=4005$$

**Figure 2:**
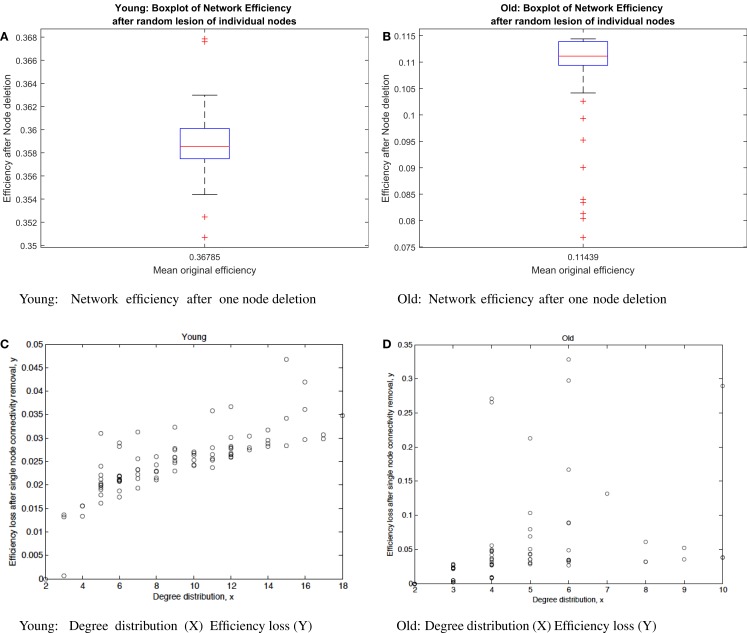
**(A)** Boxplot of network efficiency after random lesion of individual nodes in young subjects. Only a very few nodes fall outside the box whose edges are the 25th and 75th percentiles. **(B)** Boxplot of network efficiency after random lesion of individual nodes in old subjects. More nodes fall outside below the 25th percentile than in the young group. The distribution in the older group is more skewed than in the young group. **(C)** Degree distribution (x-axis) and efficiency loss or node centrality (y-axis) after single-node connectivity removal in the young condition. **(D)** Degree distribution (x-axis) and efficiency loss node centrality (y-axis) after single-node connectivity removal in the elder condition. Each dot in charts **(C,D)** represents a node with connectivity degree equals to x that upon its removal produces a variation in the network efficiency equals to y, normalized between 0 (no efficiency loss) and 1 (maximum efficiency loss). The linear regression in the young group is 0.755 and in the old group is 0.4002.

**Figure 3:**
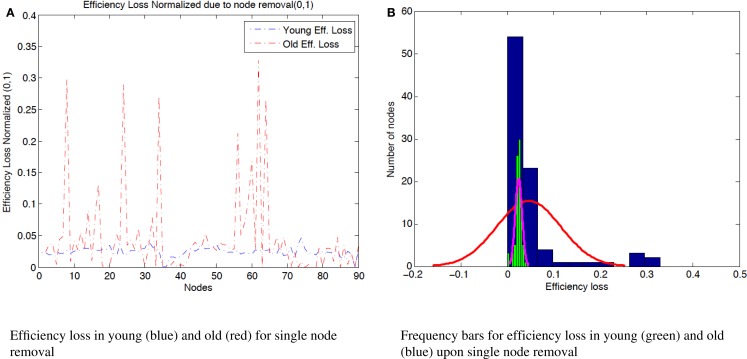
**(A)** Efficiency loss normalized (0,1) due to the removal of single nodes in both age groups. While in the young condition, there are no nodes that upon its removal, the efficiency of the resulting network deteriorates drastically; in the elder condition, there are 6 nodes that upon their removal trigger a 20% or more reduction in the network efficiency. The efficiency loss of node 8 (“Middle frontal gyrus”), 29.7%, node 24 (“Superior frontal gyrus, medial”), 28.9%, node 34 (“Median cingulate and paracingulate gyri”), 27.1%, node 56 (“Fusiform gyrus”), 21.2%, node 62 (“Inferior parietal, but supramarginal and angular gyri”), 32.8%, and node 64 (“Supramarginal gyrus”), 26.5%. **(B)** Distribution of efficiency loss after node removal in both young (green histogram) and elder groups (blue histogram). The efficiency loss in the young subjects is narrow. On the other hand, the elder subjects have a more spread distribution of efficiency values. The spread or difference between maximum and minimum efficiency loss in efficiency loss among nodes is 4.67% for young subjects and 32.87% for old subjects.

**Figure 4:**
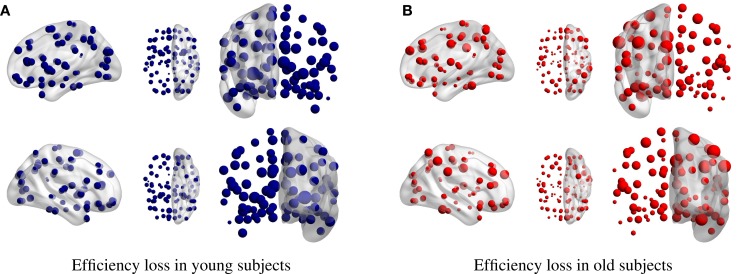
**Efficiency loss in (A) young and (B) elder condition for single-node removal**. The larger the dot size, the larger is the efficiency loss upon its removal.

We build a distribution of the efficiency measures described in Section 2 for both young and older subjects for the systematic removal of one node. Thus, in the young group, we denote *P_y_*_,90_ the distribution of networks with only one node removed, that is, *P_y_*_,90_ has 90 different networks where for each of them, one node and its connections have been deleted. The mean of the efficiency measure for *P_y_*_,90_ is 0.358. The most significant loss in efficiency occurs with the removal of node 74 (“Lenticular nucleus, putamen”) followed by node 31 (“Insula right”). The average efficiency loss in the young condition is 2.44% with a maximum of 4.67% for node 74 (“Lenticular nucleus, putamen”) and no efficiency loss for node 89 (“temporal pole: middle temporal gyrus”) (Figure 2). The rationale for the different impact in the efficiency caused by the obliteration of certain nodes can be found in the connectivity degree. In general, the nodes that, after their removal, trigger a low efficiency loss have also low connectivity degree and those that produce a more pronounced reduction of the network efficiency tend to be more connected (Figures 3 and 4).

Similarly, for the elder condition, we denote *P_e_*_,90_ the distribution of networks with only one node removed. The mean of the efficiency measure [equation (2)] for the 90 networks obtained upon single-node deletion is 0.109. As it happened in the young condition, the removal of node 89 (“Temporal pole: middle temporal gyrus”) has no effect in the efficiency. Interestingly, the removal of nodes with the lowest connectivity degree [equation (2)] has also no quantifiable effect in the network efficiency (Figure 2).

The most significant loss in efficiency occurs with the removal of node 62 (“Inferior parietal, but supramarginal and angular gyri”). After the removal of this node, the efficiency loss relative to the original network is 32.87%. This is an interesting result since node 62 is not a highly connected node and its connectivity degree is 6. Nodes 24 (“Superior frontal gyrus, medial”), 44 (“Calcarine fissure and surrounding cortex”), and 51 (“Middle occipital gyrus”) have more connections, connectivity degree as 10, and upon their deletion, the efficiency loss is not as severe as in the case of node 62. The mean efficiency loss in the elder group after the removal of a single node is 4.61% (in the young group, its 2.44%).

The connectivity degree alone is a much worse predictor of efficiency loss for old than for young subjects (Figures 3 and 4). This is in agreement with the literature of functional connectivity in healthy aging. The process of aging underlies global reorganization of brain functional networks that reflect the topological changes observed across the human lifespan (Cao et al., 2014; Song et al., 2014). Furthermore, as shown in Geerligs et al. (2015), brain networks in the elderly showed decreased modularity (less distinct functional networks) and decreased local efficiency.

### 3.2 Efficiency after Target Networks Lesioning

**Figure 5:**
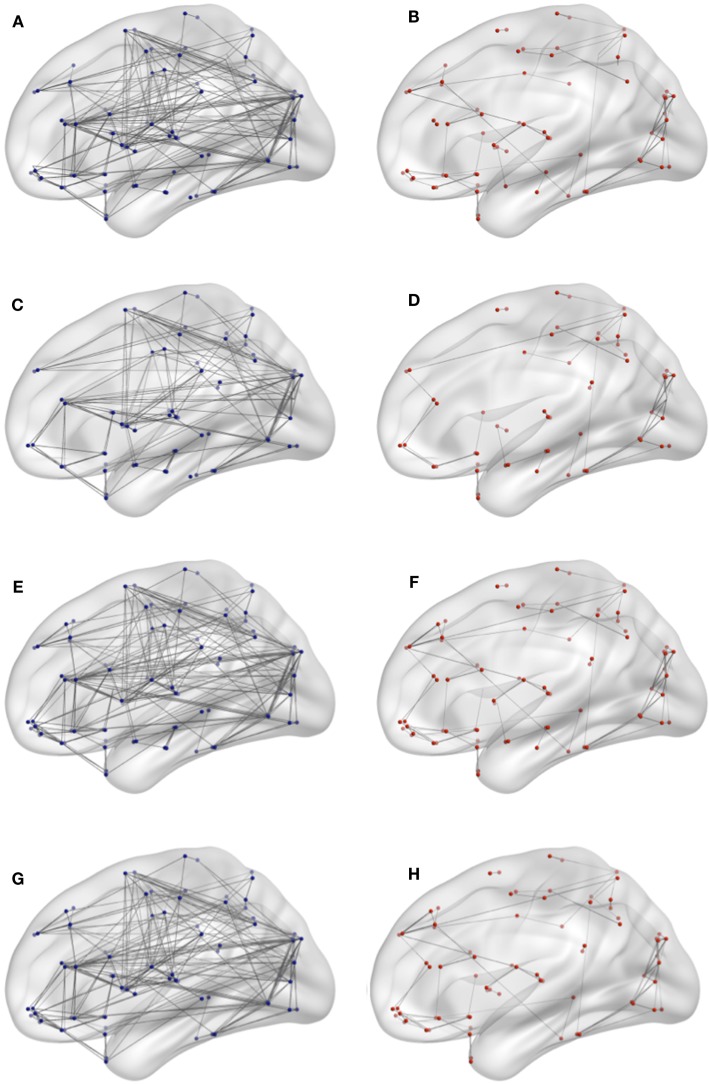
**Connectivity network for target network removal in both conditions**. **(A)** DMN lesioning in young, **(B)** DMN lesioning in elderly, **(C)** frontal lesioning in young, **(D)** limbic lesioning in elderly, **(E)** central lesioning in young, **(F)** frontal lesioning in elderly, **(G)** limbic lesioning in young, and **(H)** limbic lesioning in elderly.

So far, we have quantified the efficiency loss due to the removal of single nodes. In this section, we investigate how the efficiency measure is affected by the removal of entire networks of interest. In particular, we study the efficiency loss of the Default Mode Network (DMN), temporal lobe, frontal lobe, insula and cingulate gyrus, occipital lobe, parietal lobe, central structures, and limbic structures. The numerical results are displayed in Table 1 and brain connectivity is shown in Figure 5.

**Table 1:** **The table shows the efficiency loss after the disconnection of different brain structures in both conditions**.

Target brain structure	AAL regions	Eff. loss young (%)	Eff. loss old (%)
DMN	3, 24, 25, 26, 35, 36, 37, 68, 61, 62	19.66	61.66
Frontal lobe	1, 2, 3, 4, 5, 6, 7, 8, 9, 10, 11, 12, 13, 14, 15, 16, 17, 18, 51, 52	42.83	67.07
Temporal lobe	37, 38, 39, 40, 41, 42, 55, 56, 79, 80, 81, 82, 83, 84, 85, 86, 87, 88, 89, 90	33.56	41
Occipital lobe	43, 44, 45, 46, 47, 48, 49, 50, 51, 52, 53, 54	31.71	30.79
Parietal lobe	57, 58, 59, 60, 61, 62, 63, 64, 65, 66, 67, 68	26.65	45.64
Insula and cingulate gyrus	3, 24, 25, 26, 35, 36, 37, 68, 61, 62	18.72	36.91
Central structures (caudate nucleus, putamen, pallidum, and thalamus)	71, 72, 73, 74, 75, 76, 77, 78	23.01	3.16
Limbic structures (hippocampus, parahippocampus, and amygdala)	37, 38, 39, 40, 41, 42	9.30	1.40

*Interestingly, the reduction in efficiency is not always more pronounced in the elder condition. For example, the disconnection of the central structures (caudate nucleus, putamen, pallidum, and thalamus) triggers a larger efficiency disruption in young than in old individuals. A similar situation, larger efficiency loss in young compared old condition, also occurs with the disconnection of the limbic structures (hippocampus, parahippocampus, and amygdala) and the occipital lobe areas. The table shows the efficiency loss in both young and old groups when target networks are lesioned. The lesion consists of the obliteration of the nodes defined in the second column. The efficiency loss is larger in old adults with the exception of the occipital lobe, the central structures, and the limbic structures. The reduction of efficiency in the central structures is particularly interesting since in the old condition, it yields only a 3.16% reduction in efficiency, while in the young condition, the efficiency loss for the same lesioning yields a reduction of 23.01%*.

The DMN is commonly considered to consist of medial prefrontal cortex (AAL 23, 24, 25, and 26), posterior cingulate cortex/precuneus (AAL 35, 36/67, and 68) and bilateral inferior parietal lobule (AAL 61 and 62). The removal of the DMN in young adults triggers an efficiency loss of the 19.6%. In the elder condition, the same procedure yields an efficiency reduction of 61.66%. It is remarkable that in the elder condition, the lesioning of the DMN network, which represents the 11% of the total regions 90 regions, brings down the efficiency of the network to 61.66%. The strong efficiency reduction associated with the lesioning of the DMN in old subjects is coherent with the hypothesis that there is a decrease in activity in the DMN in aging (Koch et al., 2010). This age-based reduction in DMN activity can trigger mechanisms that compensate the loss in DMN activity with an increase in connectivity between the DMN and other networks (Damoiseaux et al., 2008). According to this hypothesis, the DMN becomes a more central network and upon the lesioning of the DMN, a dramatic efficiency loss is produced.

The removal of the frontal lobe, the parietal lobe, and the temporal lobe has a larger impact in the elder group than in the young group. Interestingly, we have identified three brain structures in which the lesioning in young individuals has a larger impact compared to old subjects. The lesioning of the occipital lobe triggers a slightly lower efficiency loss value in the old group compared to the young group. More interesting is the lesioning of the limbic structures and the central structures. The efficiency loss for these structures shows a distinct difference between young and old individuals with larger values for the former. The minor impact of the lesioning of central and limbic structures in the old condition is conforming with the literature that shows degradation of frontostratial network in aging (Salami et al., 2014) and the breakdown between the hippocampal regions and the DMN (Fjell et al., 2015). A plausible explanation is that in old subjects, the external lesioning in the simulation have been already discounted by the aging process, for example, in the form of age-related decline in the use of hippocampal relational binding networks (Rondina et al., 2015), while in the young brain, these structure are more integrated, and therefore, an external injury registers a larger efficiency loss. Furthermore, memory and attention studies show that older adults overrecruit some brain areas in an attempt to compensate for the altered function in other brain regions (Grady, 2008, 2012).

### 3.3 Efficiency after Target Networks Lesioning of Edges

To test the hypothesis that the relationship between the hippocampus and the DMN tends to break down with age, we need to lesion the edges that connect these brain structures rather than the nodes, as we have done in the previous sections. Salami et al. (2014) show that elevated hippocampal activity at rest may lower the degree to which the hippocampus interacts with other regions during memory tasks and thus results in memory deficits. However, this view is not uncontested, and in Damoiseaux et al. (2015), it is suggested that connectivity between left and right hippocampus is negatively related to age. In our study, the efficiency loss produced by the disconnection of the left and the right sides of hippocampal and parahippocampal areas does not yield a reduction of efficiency loss since these areas are not connected in the old subjects (Table 2).

**Table 2:** **Efficiency loss caused by the deletion of edges that connect brain regions in young and elder conditions**.

Network–network edges disconnection	Eff. loss young (%)	Eff. loss old (%)
DMN–DMN	0.64	0.99
HC–HC	1.43	0.45
HC–DMN	0.16	0
Frontal-striatum	0.37	0

*For example, DMN–DMN is the deletion of the edges that connect the right and the left sides of the DMN, DMN–HC the edges that connect DMN and HC, including parahippocampal areas*.

We test the asymmetry hypothesis by which brain activity shows a more balance activity among the two hemispheres with age, that is, the hypothesis predicts that in young individuals, brain activity is more asymmetric than in old individuals. The asymmetry hypothesis summons that in young individuals, the difference in efficiency loss for disconnecting the two hemispheres is expected to be larger than in the old condition. The rationale behind the hypothesis is that during aging, the brain tries to compensate the reduction of activity level, for example, in the DMN, by balancing activity across the brain. In young adults, we find that if one of the two hemispheres is entirely lesioned (all areas of one hemisphere are unreachable from the opposed hemisphere), the efficiency loss is very similar. Precisely, the efficiency loss when the left side is lesioned is 75.32%, and when the lesioning occurs in the right side, the efficiency loss is 77.01%. In old subjects, the lesioning of the right side has a more pronounced impact in the efficiency loss, 91.21% for the removal of the right side and 70.89% for the removal of the left side. This result is consistent with the HAROLD (hemispheric asymmetry reduction in older adults) model proposed by Cabeza et al. (2002). The difference in efficiency loss in old subjects after entire hemispheral disconnection is 10 times larger (~20%) than in the young subjects (~2%), which indicates that old subjects are more sensitive or less robust to unilateral disruptions because aging process tend to reduce hemispheric asymmetry. Based on these results, we hypothesize that a process of dedifferentiation may be a key mechanism to explain age-related hemispheric asymmetry reductions. As it was already mentioned in Section 3.1, the efficiency loss triggered by the disconnection of brain areas is more stereotypical (less differentiated) in the elder age group than in the young age group.

## 4 Discussion

The objective of this work is to study network robustness, i.e., resilience to perturbations, in resting-state functional connectivity networks in young and elderly conditions. The literature reviewed here suggests that graph-based network analyses are capable of uncovering system-level changes associated with aging in the resting brain. We have analyzed the functional connectivity in resting state using a perturbational approach consisting of either the systematic removal of single nodes or the removal of entire networks of interest, such as the DMN and others, and we have computed the loss in network efficiency.

Our results expand previous works on the study of robustness of structural brain networks. Interestingly, we find that the distribution of network efficiency in the young and the elder conditions show very different signatures. This is consistent with the existing evidence (Meunier et al., 2009) that both young and elderly subjects show non-random modularity and that normal aging brain is associated with changes in modularity (Song et al., 2014).

The efficiency loss in young subjects, upon the removal of single nodes is always below the 5%, while in the elder condition, the removal of individual nodes may yield a dramatic reduction of the network efficiency (maximum of 32.87%). The young adults are, thus, more robust to random deletion of single nodes. However, when the lesioning is focused in specific brain networks rather than single regions, the efficiency loss for young subjects is in occasions higher than when the same damage is done in old subjects. For example, the disconnection of the occipital lobe, limbic structures, and central structures yields larger efficiency loss in the young group. This result is compatible with the previous studies in normal healthy aging that show an increase in the functional connectivity in the sensorimotor network and a decrease in resting-state networks, including the DMN (Song et al., 2014). The continuum decrease in DMN functional connectivity found from normal aging to mild cognitive impairment and to Alzheimer’s disease (AD) can be quantitatively studied. The lowering of DMN activity is associated with better performance on attention-demanding tasks, and DMN hyperactivity is being related to negative rumination and depression (Whitfield-Gabrieli and Ford, 2012).

We replicate the common finding that older subjects present reduced functional connectivity compared to young adults (Sala-Llonch et al., 2014). Healthy normal aging is associated with cognitive decline, and the functional disconnection observed here and other studies might play an important role (Ferreira and Busatto, 2013; Dennis and Thompson, 2014). We observe the largest values of efficiency loss in old adults compared to young adults in the Default Mode Network and the frontal lobe (Table 1). This is consistent with the compensation hypothesis in healthy aging, which states that older adults brains compensate for the overall functional deficits by increasing the activity levels in frontal regions, as part of a reorganization process mediated by healthy normal aging (Cabeza et al., 2002; Park and Reuter-Lorenz, 2009). In this view, the DMN is a highly susceptible system in healthy aging (Onoda et al., 2012; Betzel et al., 2014).

Can efficiency loss be used as a predictor of brain network differential activity? Vergun et al. (2013) applied a Support Vector Machine (SVM) linear classifier to rs-fMRI data in order to compare age-related differences in four of the major functional brain networks: the default, cingulo-opercular, frontoparietal, and sensorimotor. The classifier was able to detect “connectivity hubs,” or nodes with the most significant features that influenced age classification. More work is, however, needed in order to properly address the compatibility of informational efficiency measures with non-parametric classifiers.

A natural continuation of this work is to incorporate a translational outlook to, for example, investigate whether hubs of human brain networks are more likely to be anatomically abnormal than non-hubs in brain disorders (Crossley et al., 2014). Informational efficiency measures may also shed light on the dynamics and control of resting-state networks in mental disorders. This perturbational approach can also be extended to study the interplay between network efficiency and brain metabolic demand, aiming to identify pathological signatures for early diagnosis in neurodegenerative disorders. The network dynamics associated with different conditions – normal healthy aging, mild cognitive impairment, Alzheimer’s disease, etc. – can be simulated with the same or similar type of functional intervention proposed here. Interventions other than disconnecting regions of interest or entire subnetworks from the whole brain may include stress simulations induced by impairment of structural, functional, or both connectivity patterns in multimodal imaging models. The computational lesioning of brain foci holds promise for systemic understanding of compensatory and other network mechanisms, e.g., cascade and contagion effects, under normal and pathological conditions.

## Author Contributions

JG-R, YL, QW, and JW designed the study, developed the methodology, performed the analysis, and wrote the manuscript. JG-R, YL, and QW collected the data.

## Conflict of Interest Statement

The authors declare that the research was conducted in the absence of any commercial or financial relationships that could be construed as a potential conflict of interest.

## Footnotes

^1^http://www.fil.ion.ucl.ac.uk/spm/

^2^http://restfmri.net/forum/index.php

^3^http://imaging.mrc-cbu.cam.ac.uk/imaging/Templates
